# The Identification of Proteomic Signatures Associated with Alkaline Tolerance in the Skin Mucus of Crucian Carp (*Carassius auratus*)

**DOI:** 10.3390/ijms252111618

**Published:** 2024-10-29

**Authors:** Zhipeng Sun, Jing Huang, Xiaofeng Zhang, Yumei Chang, Guo Hu

**Affiliations:** Key Laboratory of Freshwater Aquatic Biotechnology and Breeding of Ministry of Agriculture and Rural Affairs, Heilongjiang River Fisheries Research Institute of Chinese Academy of Fishery Sciences, Harbin 150070, China; sunzhipeng@hrfri.ac.cn (Z.S.); huangjing@hrfri.ac.cn (J.H.); zhangxiaofeng@hrfri.ac.cn (X.Z.); changyumei@hrfri.ac.cn (Y.C.)

**Keywords:** *Carassius auratus*, proteomics, alkali-tolerant fish, biomarker

## Abstract

The skin is covered by a protective mucus layer, which is essential to the innate defense mechanism of fish. Investigating the response of skin mucus to various toxic stresses is crucial for enhancing its ability to tackle environmental challenges and developing strategies to mitigate toxic effects. Alkalinity stress assays (50 mmol/L NaHCO_3_) were conducted on crucian carp (*Carassius auratus*) from Lake Dali Nur (pH = 9.6) and Ping Xiang red crucian carp from freshwater (pH = 7) over 7 days. The expression of skin mucous proteins was analyzed using the liquid chromatography (LC)-spectrometry (MS)/MS Analysis-Data-independent acquisition (DIA) mode. A total of 12,537 proteins were identified across 20 samples from four groups, with 12,025 quantified. In the alkaline water population, high alkali stress resulted in the up-regulation of 139 proteins and the down-regulation of 500 proteins. In contrast, the freshwater population showed an increase in 112 proteins and a decrease in 120; both populations had a total of 23 genes up-regulated and 21 down-regulated. The protein regulatory network for the alkaline water group included 3146 pairwise interactions among 464 nodes, with only 20 being differentially expressed proteins. Conversely, the freshwater group’s network comprised just 1027 specific interactions across 337 nodes, with 6 corresponding to differentially expressed proteins. A common protein regulatory network responding to high alkali stress was extracted and visualized for both populations. Based on their regulatory relationships and expression levels, these proteins are hypothesized to play similar roles under high alkali stress. Notably, the alpha-globin fragment and keratin type I cytoskeletal 13-like proteins showed markedly up-regulated expression, with the alpha-globin fragment increasing nearly a thousandfold from an extremely low level. This suggests it could serve as a potential biomarker for alkali tolerance, warranting further investigation.

## 1. Introduction

China ranks third globally in terms of saline–alkali land area, covering an extensive 46 million hectares [1]. Currently, only about 2 percent of this land is utilized for aquaculture [2]. The primary ionic components present in inland saline–alkali waters are carbonate and bicarbonate ions. The high alkalinity and pH levels adversely affect the growth, survival, and reproductive capacity of fish, presenting significant challenges for aquaculture practices [3]. As aquatic organisms, fish are continuously subjected to a variety of stressors that lead to the development of complex immunomodulatory networks aimed at ensuring effective immune responses and maintaining homeostatic balance [4]. A thorough assessment of alkali tolerance traits is crucial for genetic selection; moreover, identifying biomarkers associated with alkali tolerance enables the precise evaluation of their impacts on growth performance, feed utilization efficiency, immune health, product quality, and safety, as well as exacerbation of fish diseases.

Currently, it is widely acknowledged that highly alkaline water directly influences the equilibrium between gaseous NH_3_ and NH_4_^+^ in fish, potentially giving rise to NH_3_ retention and the augmentation of endogenous NH_3_ [5]. Accordingly, reduced levels of blood and water ΔPNH_3_ (ammonia partial pressure gradient) might hamper the efficacy of ammonia excretion. Ineffective ammonia excretion is capable of leading to an excessive accumulation of endogenous ammonia, exerting an influence on exercise performance and blood indicators, modifying energy mobilization and metabolic patterns, and interfering with ion regulation [6]. In addition to environmental factors that impede ammonia excretion and have an impact on the gill boundary buffer potential gradient, effective excretion mechanisms are indispensable for preventing endogenous ammonia poisoning [7]. For instance, enhancing the body’s tolerance to ammonia represents a crucial strategy for naked carp (*Gymnocypris przewalskii*) to adapt to a highly alkaline environment. Distinct from other species, its muscles and brain can tolerate high concentrations of ammonia without depolarization [8]. The up-regulation of specific transporters and the activation of diverse ammonia excretion mechanisms are of significant importance for alkali-tolerant fish to survive in an alkaline environment. The Lake Magadi tilapia (*Alcolapia grahami*) is capable of converting all toxic ammonia to non-toxic urea nitrogen through the ornithine urea cycle enzyme [9], while elasmobranch converts it to urea for utilization as nitrogen energy via the ornithine urea cycle pathway.

Crucian carp (*Carassius auratus*) is a widely distributed species across Eurasia, inhabiting diverse environments [10]. It holds significant importance as a farmed fish in numerous Asian countries, particularly China [11]. It is notable for being one of the few freshwater aquaculture species capable of tolerating high levels of alkaline stress [12]. According to the data of the China Fishery Statistical Yearbook, China’s aquaculture production exceeded 2840 thousand tons in 2023. Focusing research on crucian carp presents significant practical advantages for optimizing China’s utilization of alkaline water resources. Similarly to the other fish species mentioned above, current knowledge of crucian carp’s response to alkaline and salt osmotic challenges mainly concerns its plasma and tissue metabolism and osmotic pressure regulation [13,14]. However, limited research has been conducted on skin mucus to investigate the responses of crucian carp to salt and alkali stress thus far. Unlike mammalian skin, the skin of teleost fish consists of mucosal tissue that reacts similarly to the intestinal mucosa in response to stimuli [15,16]. A protective layer of mucus envelops their scaly skin; this secretion serves as a critical component in the primary line of defense. Research into how skin mucus responds to toxic stresses is essential for enhancing coping mechanisms and developing strategies aimed at mitigating toxic effects [17,18]. Considering modifications in the composition of skin mucus during prolonged exposure to alkali and salt stress within commercial cultures in saline waters, we selected two populations of crucian carp: one from an extremely alkaline environment and another from freshwater. Lake Dali Nur, located on the eastern Inner Mongolia Plateau in North China (116°25′–116°45′ E, 43°13′–43°23′ N), is a volcanic barrier lake characterized by high concentrations of alkali carbonate. This phenomenon arises from lower evaporation rates compared to precipitation and inflow [19]. In this extreme aquatic environment, where alkalinity levels can reach up to 50 mmol/L NaHCO_3_ (pH = 9.6), crucian carp not only survive but also thrive; they are among the few fish species capable of reproducing and growing under such challenging conditions. Consequently, they play a crucial role in both ecological diversity and local livelihoods [20]. Another population is the red crucian carp native to the Pingxiang region of Jiangxi Province, China [21]. This population flourishes in freshwater ecosystems with a pH of approximately 7, yet its tolerance to highly alkaline conditions remains largely unexplored. We investigated the potential use of mucus composition as an indicator of physiological response by assessing how these two populations of crucian carp respond to alkali-salt stresses and measuring proteomic alterations in their mucus.

This study aimed to examine the alterations in the protein composition of skin mucus among freshwater and alkaline water populations of crucian carp subjected to elevated NaHCO_3_ alkalinity stress. To illuminate the functional roles of genes and identify potential biomarkers associated with these alterations in protein composition, we carried out Gene Ontology (GO) functional annotation, Kyoto Encyclopedia of Genes and Genomes (KEGG) enrichment analysis, and regulatory network analysis. Our findings enhance the understanding of how crucian carp respond to environmental alkalinity through the analysis of skin mucus. This research may offer significant benefits for conservation biology studies and aquaculture in inland saline–alkali water bodies, as well as potential applications in genetic breeding programs.

## 2. Results

### 2.1. Principal Component and Correlation Analysis

**Figure 1:**
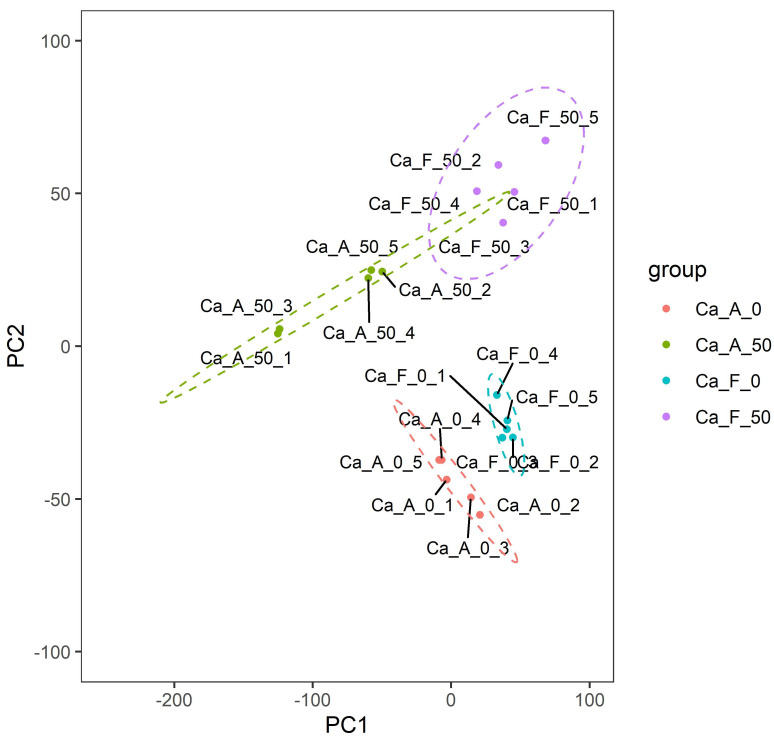
Principal component analysis (PCA) of 20 sequenced individuals in four groups. The dots imply diverse specimens, and the colors denote distinct experimental groups. The circles of multiple hues evince the clustering among disparate groups, and the magnitude of the circles typically reflects the degree of dispersion of the samples within the group. The interpretation of the group name abbreviations: “Ca” represents the Latin abbreviation for *Carassius auratus*; “A” stands for the alkaline water population; “F” stands for the freshwater population; “0” indicates that no NaHCO_3_ is added to the aquaculture water; “50” indicates the addition of 50 mmol/L NaHCO_3_ in the aquaculture water; and 1 to 5 are the number of sequenced individuals randomly assigned within the group.

**Figure 2:**
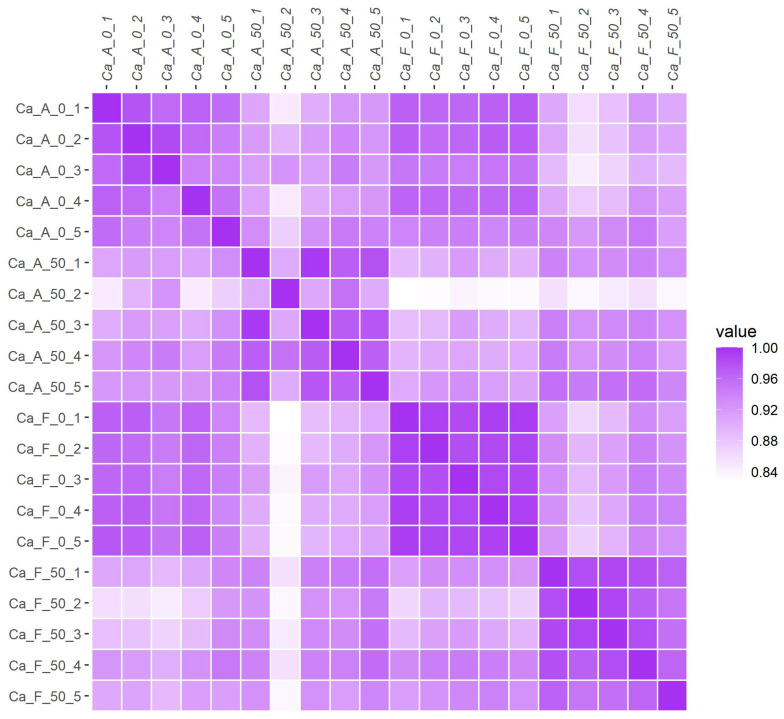
Overview of correlation analysis of expression patterns within and between groups for the 20 sequenced individuals. Correlation coefficients from 0 to 1 are shown in blank to dark purple, showing 5 sequenced individuals in 4 groups. The interpretation of the group name abbreviations: “Ca” represents the Latin abbreviation for *Carassius auratus*; “A” stands for the alkaline water population; “F” stands for the freshwater population; “0” indicates that no NaHCO_3_ is added to the aquaculture water; “50” indicates the addition of 50 mmol/L NaHCO_3_ in the aquaculture water; and 1 to 5 are the number of sequenced individuals randomly assigned within the group.

A total of 12,537 proteins were identified across 20 samples from four distinct groups, with 12,025 proteins quantified (Table S1). Principal component analysis (PCA) was performed, enabling us to observe the relative positions and distributions of the sample groups within the principal component space. This analysis revealed significant differences among the groups (Figure 1). Through this graph, we can discern the relative positions and distributions of the four groups of samples in the principal component space. Correlation analysis further demonstrated remarkable parallelism within each sample group (Figure 2).

### 2.2. Identification and Cluster Analysis of Differentially Expressed Proteins

**Figure 3:**
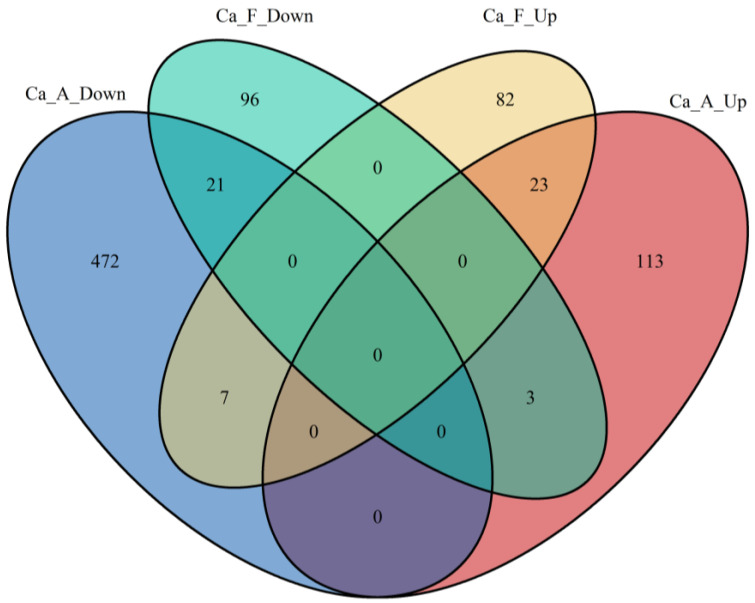
The number of differentially expressed proteins in and among all 4 groups in the high alkali stress experiment. Intersection is the number of differentially expressed proteins shared by groups. The interpretation of the group name abbreviations: “Ca” represents the Latin abbreviation for *Carassius auratus*; “A” stands for the alkaline water population; and “F” stands for the freshwater population; “Up” represents proteins that are up-regulated; and “Down” represents down-regulated proteins.

**Figure 4:**
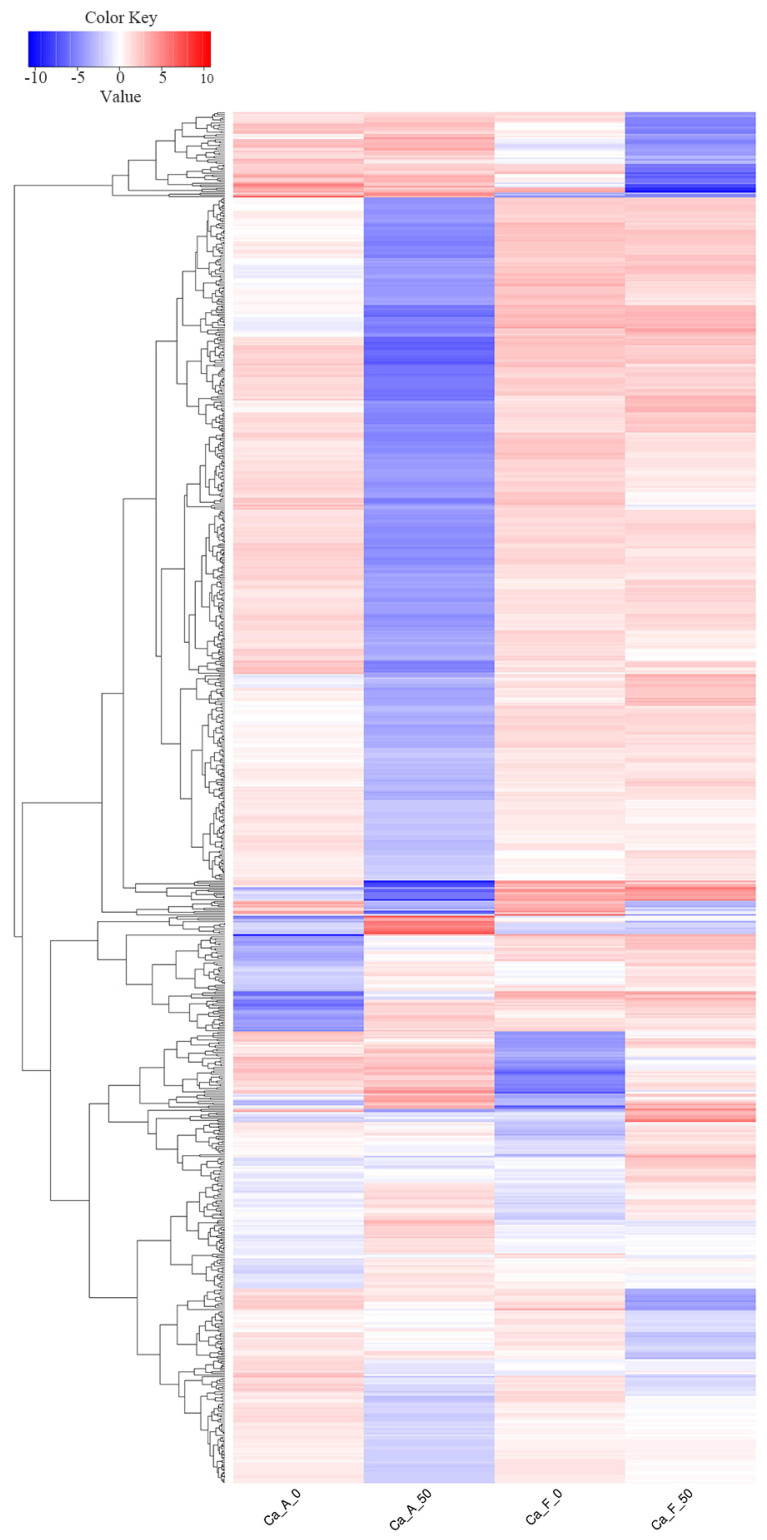
The expressional profiles of the differentially expressed proteins in and among all 4 groups in the high alkali stress experiment. The interpretation of the group name abbreviations: “Ca” represents the Latin abbreviation for *Carassius auratus*; “A” stands for the alkaline water population; “F” stands for the freshwater population; “0” indicates that no NaHCO_3_ is added to the aquaculture water; and “50” indicates the addition of 50 mmol/L NaHCO_3_ in the aquaculture water.

In alkaline water populations, a comprehensive analysis of differential protein expression revealed that 139 proteins were significantly up-regulated, while 500 proteins were significantly down-regulated following exposure to high alkali stress. In contrast, freshwater populations exhibited a comparative assessment showing that 112 proteins were up-regulated and 120 were down-regulated. Notably, in both populations, a total of 23 genes demonstrated up-regulation, and 21 showed down-regulation (Figure 3). To elucidate the expression patterns of these differentially expressed proteins under varying conditions, we meticulously illustrated the clustering of these proteins. Following high alkali stress, the differentially expressed genes exhibited markedly distinct clustering patterns in alkaline compared to freshwater populations (Figure 4).

### 2.3. Functional Annotation Analysis of Differentially Expressed Proteins

**Figure 5:**
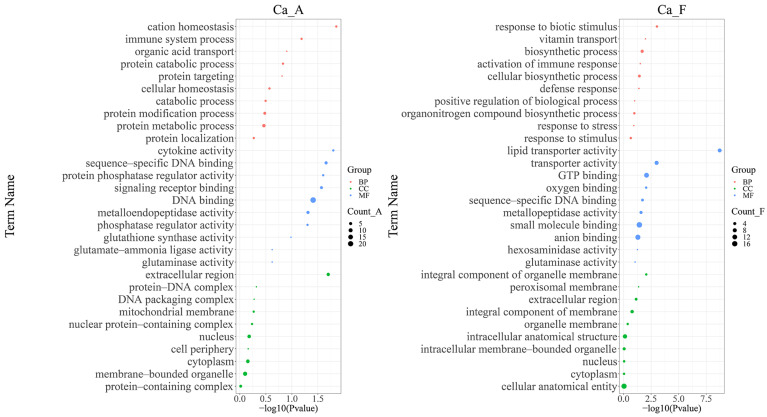
Gene Ontology enrichment analysis of differentially expressed proteins for each population in high alkali stress experiment. The interpretation of the group name abbreviations: “Ca” represents the Latin abbreviation for *Carassius auratus*; “A” stands for the alkaline water population; and “F” stands for the freshwater population. Red dots correspond to GO terms associated with biological processes (BP), blue dots correspond to GO terms associated with molecular functions (MF), and green dots correspond to GO terms associated with cellular components (CC). The size of each dot represents the number of genes involved in the respective GO term. The X-axis represents the *p*-value of the topGO enrichment analysis, which was transformed using −log10 (*p*). The Y-axis represents the GO terms themselves.

In the alkaline water population, a total of 639 differentially expressed proteins were significantly enriched in several Gene Ontology (GO) categories: cation homeostasis (GO:0055080, biological process), immune system process (GO:0002376, biological process), cytokine activity (GO:0005125, molecular function), sequence-specific DNA binding (GO:0043565, molecular function), extracellular region (GO:0010468, cellular component), and protein–DNA complex formation (GO:0032993, cellular component). Furthermore, three GO definitions related to glutathione metabolism were annotated (Figure 5 Ca_A).

In freshwater populations, 232 differentially expressed proteins were predominantly enriched in the following categories: immune system process (GO:0002376, biological process), response to biotic stimulus (GO:0009607, biological process), lipid transporter activity (GO:0005319, molecular function), metalloendopeptidase activity (GO:0004222, molecular function), integral component of organelle membrane (GO:0031301, cellular component), and protein–DNA complex formation (GO:0032993, cellular component). Additionally noted are three GO definitions pertaining to glutathione metabolism (GO:0004356, glutamine synthetase activity; GO:0004363, GO:0004359, glutaminase activity; glutathione synthase activity). A similar analysis featuring distinct GO entries is illustrated in Figure 5 Ca_F.

**Figure 6:**
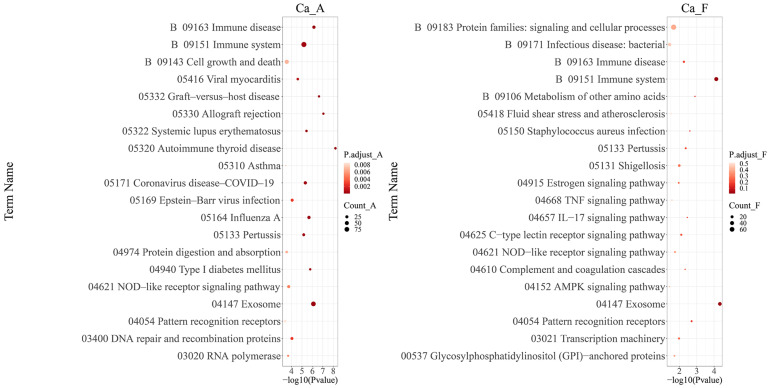
The KEGG pathway enrichment analysis of differentially expressed proteins for each population in a high alkali stress experiment. The interpretation of the group name abbreviations: “Ca” represents the Latin abbreviation for *Carassius auratus*; “A” stands for the alkaline water population; and “F” stands for the freshwater population. The dot size represents the number of genes involved in the KEGG term; the X-axis is the *p*-value of the KEGG enrichment analysis, with −log10 transformation, −log10 (*p*), while the Y-axis is KEGG terms.

The KEGG enrichment pathways for these populations under high alkali stress demonstrated significant differences. However, four pathways—‘B 09151 Immune system’, ‘B 09163 Immune disease’, ‘04147 Exosome’, and ‘04621 NOD-like receptor signaling pathway’—exhibited notable consistency. Furthermore, the ‘B 09151 Immune system’ displayed a significantly higher abundance of differentially expressed proteins (Figure 6).

### 2.4. Regulatory Network Analysis and Identification of Potential Alkaline Tolerance-Associated Biomarkers

**Figure 7:**
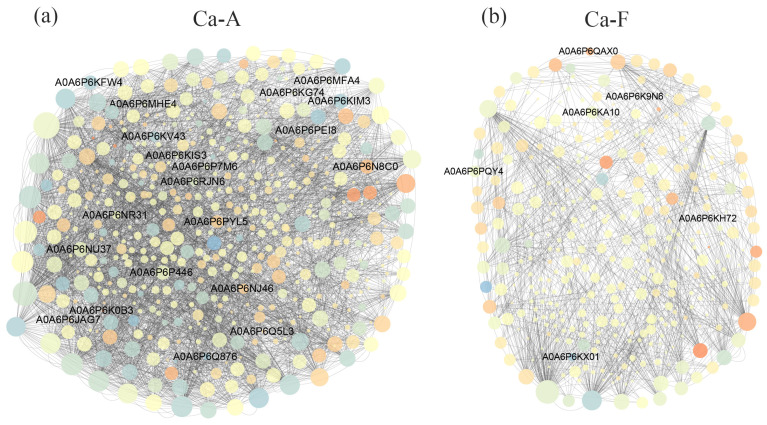
Gene regulatory network analysis of differentially expressed proteins for each population in the high alkali stress experiment. (**a**) shows the regulatory network of proteins identified in the skin mucus of the alkaline water population; (**b**) shows the regulatory network of proteins identified in the skin mucus of the freshwater population. The interpretation of the group name abbreviations: “Ca” represents the Latin abbreviation for *Carassius auratus*; “A” stands for the alkaline water population; and “F” stands for the freshwater population. The size of the nodes is indicative of the number of connections among them; red nodes represent up-regulated expression, whereas blue nodes indicate down-regulated expression. The color intensity serves as a quantitative measure of the magnitude of differential expression. The node names labeled on the figure are differentially expressed proteins.

**Figure 8:**
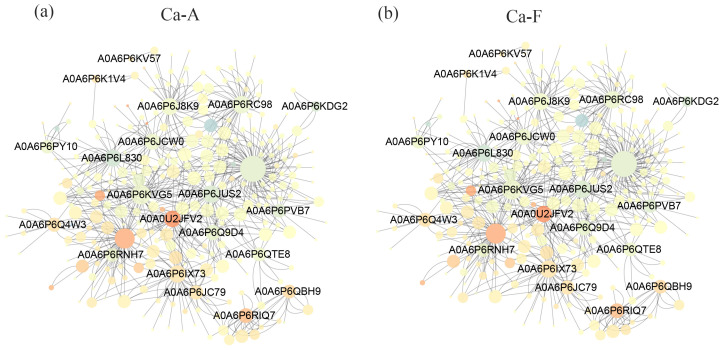
Gene regulatory network analysis of differentially expressed proteins shared in both populations in the high alkali stress experiment. (**a**) shows the regulatory network of proteins with expression level identified in the skin mucus of the alkaline water population shared with freshwater population; (**b**) shows the regulatory network of proteins with expression level identified in the skin mucus of the freshwater population shared with alkaline water population. The interpretation of the group name abbreviations: “Ca” represents the Latin abbreviation for *Carassius auratus*; “A” stands for the alkaline water population; and “F” stands for the freshwater population. The size of the nodes is indicative of the number of connections among them; red nodes represent up-regulated expression, whereas blue nodes indicate down-regulated expression. The color intensity serves as a quantitative measure of the magnitude of differential expression. The node names labeled on the figure are differentially expressed proteins.

In the alkaline water population, the protein regulatory network consisted of 3146 pairwise regulatory interactions involving 464 nodes, among which 20 were identified as differentially expressed proteins (Figure 7a). In contrast, the protein regulatory network of freshwater populations comprised only 1027 specific pairwise interactions distributed across 337 nodes, with merely 6 corresponding to differentially expressed proteins (Figure 7b). The reconstructed protein regulatory network, which is based on a shared regulatory relationship between the two populations, includes 646 paired interactions involving 259 distinct nodes (Figure 8).

Among these node proteins, twenty are also classified as differentially expressed; seven exhibited consistent up-regulation in both populations, eleven demonstrated consistent down-regulation, and two displayed inconsistent expression patterns across the two populations. Among these node proteins, the most promising positive biomarkers identified are A0A0U2JFV2 and A0A6P6RIQ7, corresponding to alpha-globin and keratin type I cytoskeletal 13-like, respectively (Table 1).

## 3. Discussion

The composition of fish skin mucus is intricate and multifaceted. It is primarily produced by mucin-producing cells, which secrete it onto the surface of the skin, with mucin serving as the predominant component. Furthermore, this mucus encompasses a variety of enzymes and immune factors, including lysozyme, cathepsin, esterase, metalloproteinase, immunoglobulin, lectin, interferon, calcetin, complement proteins, antimicrobial peptides, histones, and ribosomal proteins [15,22]. This study identified over 12,000 distinct proteins present in the mucus on the skin surface of crucian carp. These findings underscore the complexity and diversity of active substances contained within fish skin mucus.

Typically, alkaline tolerance is assessed by measuring the survival rate after a 96-h exposure to alkaline conditions or by determining the semi-lethal concentration during the period of alkaline stress [23,24]. However, these conventional methods do not accurately reflect long-term tolerance to alkaline environments and significantly differ from actual commercial aquaculture settings and cycles. Therefore, in this study, we extended the traditional 96-h stress period to 7 days in order to better simulate the real conditions encountered in commercial aquaculture.

In this investigation, principal component analysis revealed that mucus from alkaline and freshwater populations exhibited distinct characteristics when subjected to high alkalinity stress (50 mmol/L NaHCO_3_). These findings suggest a strong genetic basis for the tolerance of both populations to sodium bicarbonate alkali salt stress. Furthermore, correlational analysis of protein expression demonstrated a significant degree of consistency within each group, thereby establishing a solid foundation for the reliability of subsequent findings. This is consistent with our previous studies on Amur Ide (*Leuciscus waleckii*) [2,25,26]. The number of differentially expressed proteins in the alkaline population (639) was significantly greater than that observed in their freshwater counterparts (232). These findings suggest that alkaline populations possess enhanced tolerance against alkali stress as a result of prolonged environmental natural selection. This implies that alkali tolerance is supported by both physiological and genetic factors, thus positioning genetic selection as a promising strategy for future research endeavors.

Researchers previously conducted a series of studies to investigate the high alkaline tolerance of crucian carp. The results indicated that saline–alkali habitat is a key factor influencing the transformation of ammonia metabolism in these fish [13,14,20,27]. An increase in sodium bicarbonate alkalinity leads to elevated concentrations of exogenous ammonia nitrogen, which hinders the metabolism and transformation of endogenous nitrogen, ultimately affecting the physiological levels of ammonia excretion [28]. The glutamate metabolic pathway plays a crucial role in promoting ammonia excretion and detoxification in crucian carp [13,14]. In this study, we identified significant concentrations of differentially expressed proteins associated with glutamine synthetase activity in both alkaline and freshwater populations. Additionally, activities related to glutaminase, glutathione synthase, and other Gene Ontology (GO) terms corroborated our previous experimental findings, thereby enhancing the credibility of this study. Results from GO and KEGG enrichment analyses revealed that many differentially expressed proteins are linked to immune system functions. These proteins are responsible for recognizing, eliminating, and removing invading pathogens and harmful substances; they play an essential role in resisting pathogen invasion while maintaining homeostasis. This observation aligns with findings from other researchers regarding the abundance of immune-related components present in skin mucus [29,30]. Current research on fish skin mucus proteomics has highlighted significant differences in protein composition across various species [31]. Further investigation is warranted to fully elucidate these differences. Nonetheless, our study on crucian carp skin mucus proteomics contributes valuable insights into establishing mucosal protein profiles that will aid in identifying additional immune-related proteins found within skin mucus. This work could provide more concrete data for understanding the immune-related roles played by skin mucus.

In the examination of the protein expression regulatory network, it becomes evident that the alkaline population exhibits a markedly intricate network topology characterized by a plethora of pairwise interactions and an extensive reach. In contrast, freshwater populations display a relatively simplistic network structure, with significantly fewer notable nodal proteins present within their framework. This is consistent with previous results from Amur Ide (*Leuciscus waleckii*) [32,33]. Notably, the pH measured in this study was 9.12 ± 0.35, which is slightly lower than the typical water alkalinity of its long-term habitat (pH = 9.6). The divergence in network topology between these two populations intuitively suggests that inhabitants of alkaline waters have activated a substantial array of functional genes in response to elevated alkali stress; conversely, their freshwater counterparts demonstrate considerably diminished efficacy in responding to such stressors. From both populations, we extracted and visualized a common protein regulatory network relevant to high alkali stress responses. Based on their regulatory relationships and expression levels, these proteins are posited to fulfill analogous roles when confronted with high alkali conditions. Among them, alpha-globin fragments and keratin-type I cytoskeletal 13-like proteins exhibit pronounced up-regulation; particularly noteworthy is the alpha-globin fragment, whose expression has surged from an exceedingly low baseline—an increase approaching a rise of over thousands of folds. Globin, a class of respiratory proteins capable of reversibly binding oxygen through iron-porphyrin rings, is widely distributed across bacteria, fungi, plants, and animals. It exhibits significant structural and functional diversity [34]. The mechanism underlying alkaline tolerance involves the accumulation of ammonium ions alongside its efficient transport exclusions; this process is energy-intensive and leads to considerable increases in oxygen consumption as metabolic alterations occur [35,36]. Alpha-globin stands out as an essential functional protein, warranting further investigation into its physiological mechanisms underpinning tolerance to high alkalinity [37,38]. The expression level of keratins is generally positively correlated with the degree of inflammation present in the body [39]. An increase in the expression level of this protein indicates that alkalinity stress leads to a significant elevation in inflammatory responses. It is hypothesized that this alteration represents a broad-spectrum mechanism underlying environmental stress rather than being specific to alkali tolerance.

## 4. Materials and Methods

### 4.1. Animal Ethics Statement

All animal experimental procedures in this study were carried out in accordance with the guidelines for the care and use of experimental animals of Heilongjiang River Fisheries Research Institute, Chinese Academy of Fishery Sciences. The animal experiments were reviewed and approved by the Experimental Animal Welfare and Ethics Committee of Heilongjiang River Fisheries Research Institute, Chinese Academy of Sciences (Approval code: 2015-02-28).

### 4.2. Fish Samples and NaHCO_3_ Alkalinity Stress Experiment

In the present study, a freshwater control group and an alkalinity treatment group (50 mmol/L, NaHCO_3_) were, respectively, established for the alkaline population originating from Lake Dali Nur with a pH value of 9.6, and the freshwater population of red crucian carp originating from Pingxiang, Jiangxi Province, where the water environment has a pH value of 7. To explore whether alkaline tolerance possesses a genetic basis, these alkaline populations were cultivated in freshwater environments commencing with larvae. A total of 150 individuals, of which 75 were from the alkaline population, and 75 were from the freshwater population, exhibiting a body weight of (67.27 ± 16.42) g and a body length of (12.07 ± 1.55) cm, were randomly selected for temporary feeding in an indoor circulation tank over the course of one week. These fish demonstrated strong activity, were healthy, and were free from injury. During the period of temporary maintenance, appropriate daily feeding was administered, continuous oxygenation was ensured, and a water change equivalent to one-third of the tank volume was performed every other day. The experimental water maintained an average dissolved oxygen level of (7.51 ± 0.65) mg/L, with a temperature of approximately (17.84 ± 2.73 °C) (The inland saline–alkali water areas of North China and Northeast China have an average temperature of about 18 °C during the seasons without ice), measured alkalinity at (0.51 ± 0.04) mM, and salinity recorded at (0.1 ± 0.02) g/L. Food was withheld for a duration of 48 h prior to the alkalinity tolerance test. A total of 120 active, healthy, and injury-free individuals, with 30 in each group, were employed for the follow-up alkali tolerance tests. NaHCO_3_ (analytical purity, Tianjin Kaitong Chemical Reagent Co., Ltd., Tianjin, China) was adopted to configure the corresponding alkalinity. The alkaline population and freshwater population were tested, each consisting of 60 individuals. Moreover, the two populations were randomly selected to constitute two groups, each containing 30 individuals. A total of thirty fish from each group were placed in a 400 L indoor circulating aquarium (177 cm × 57 cm × 40 cm) for a period of seven days for an alkalinity stress assay. Throughout the experiment, no feeding was implemented; nonetheless, oxygenation persisted, half of the water volume was exchanged daily, and various water quality parameters were monitored. The NaHCO_3_ alkalinity was evaluated through titration, pH levels were registered using a pH meter (pH 400 portable pH meter from Shanghai Peiru Instrument Co., Ltd., Shanghai, China), dissolved oxygen levels were gauged with an AZ 8371 device (from Hengxin Industrial Co., Ltd., Taichung, China), and temperature readings were obtained using a standard thermometer. The experimental water contained dissolved oxygen levels averaging (7.51 ± 0.65) mg/L and temperatures of approximately (17.84 ± 2.73 °C); meanwhile, the measured alkalinity of the control group was (0.51 ± 0.04) mM compared to (50.65 ± 1.35) mM in the experimental group. The corresponding pH values were recorded as 7.79 ± 0.19 versus pH 9.12 ± 0.35, while salinities were measured at (0.1 ± 0.02) g/L versus (3.6 ± 0.52) g/L, respectively.

### 4.3. Collection of Skin Mucus Samples

Subsequent to the 7-day alkali stress experiment, five fish from each group were carefully selected and anesthetized with MS-222 (Sigma, St. Louis, MO, USA). The surfaces of the test specimens were meticulously cleaned with double-distilled water, a procedure that was repeated 2 to 3 times before being placed in appropriately sized Petri dishes. Subsequently, 10 mL of double-distilled water at 4 °C was added and allowed to stand for 8–10 min. A sterilized microscope slide was then utilized to gently scrape the mucus from the fish’s snout along its body towards the tail, performing this scraping motion back and forth three times. The collected mucus was transferred into a 5 mL cryogenic tube, frozen in liquid nitrogen, and stored in an ultra-low temperature freezer at −80 °C.

### 4.4. Total Protein Extraction and Peptide Digestion

The skin mucus samples were individually homogenized in liquid nitrogen and subsequently lysed with Sodium deoxycholate (SDT, containing 100 mM NaCl) (Sigma, USA) along with a Dithiothreitol (DTT) concentration of 1/100 (Sigma, USA). Following this, ultrasonication was performed on ice for five minutes. After an incubation period of 8–15 min at 95 °C, the samples underwent an ice bath treatment for two minutes prior to centrifugation at 12,000× *g* for fifteen minutes at 4 °C. The resulting supernatant was then alkylated with a precise amount of iodoacetamide (IAM) (Sigma, USA) in the dark at room temperature for one hour. Subsequently, the samples were thoroughly mixed with four volumes of precooled acetone via vortexing and incubated at −20 °C for no less than two hours. They were subjected to another round of centrifugation at 12,000× *g* for fifteen minutes at 4 °C to collect the precipitate. Finally, after washing with one mL of cold acetone (Beijing Chemical Plant, Beijing, China), the pellet was completely dissolved in Dissolution Buffer (DB buffer) (Sigma, USA). For the purpose of proteomic analysis, proteins extracted from each sample underwent trypsin digestion using the filter-aided proteome preparation method [40]. The resulting peptides were desalted utilizing a C18 Cartridge (Empore^TM^ SPE Cartridges C18 (standard density), bed I.D. 7 mm, volume 3 mL, Sigma, USA), and subsequently freeze-dried before being resuspended in 40 μL of a 0.1% formic acid solution (Thermo Fisher Scientific, Waltham, MA, USA).

### 4.5. LC-MS/MS Analysis-DIA Mode

The approach employed for the analysis using liquid chromatography–tandem mass spectrometry (LC-MS/MS) involved examining peptides from each sample with an Orbitrap^TM^ Astral^TM^ mass spectrometer (Thermo Fisher Scientific, Waltham, MA, USA), which was integrated with a Vanquish Neo system liquid chromatography (Thermo Fisher Scientific, Waltham, MA, USA) operating in DIA mode. Precursor ions were detected across a mass range of 380–980 m/z, achieving an MS1 resolution of 240,000 at 200 *m*/*z*, alongside a normalized AGC target set to 500% and a maximum ion time of 5 ms. During the MS2 scanning phase within the DIA mode, a total of 299 windows were established with an isolation window measuring 2 m/z; HCD collision energy was calibrated to 25 eV while maintaining a normalized AGC target of 500% and limiting the maximum IT to 3 ms. The data obtained from DIA analysis was processed using DIA-NN version 1.8.1 at a stringent confidence level of 99% for protein identification purposes. This objective was achieved by implementing a false discovery rate threshold not exceeding ≤1%. Key parameters in the software included trypsin as the enzyme choice, allowing one missed cleavage at most. Carbamidomethyl (C) served as the fixed modification applied, while dynamic modifications consisted of oxidation (M) and acetylation at the Protein N-terminus. Novogene Co., Ltd. (Beijing, China) was entrusted to furnish technical services pertaining to protein extraction, quantification, detection, enzyme digestion and desalination, fraction separation, as well as mass spectrometry detection.

### 4.6. Bioinformatics Analysis

In this study, the raw data files underwent thorough examination and processing using the DIA-NN library search software, version 1.9 [41]. To identify homologous sequences, protein sequences were analyzed locally with NCBI BLAST+ client software, version 2.11.0 (NCBI, Bethesda, MD, USA) and InterProScan 5.66–98.0 (European Molecular Biology Laboratory–European Bioinformatics Institute, Hinxton, Cambridge, UK) [42,43]. Following this step, a wide range of bioinformatics tools and R language packages were utilized for a comprehensive analysis of proteomic data. Initially, expression values for all proteins were modified by adding +100; thereafter, principal component analysis (PCA) was performed utilizing R’s factoextra and FactoMineR packages to pinpoint the main sources of variation within the dataset [44,45,46]. Correlation analysis was then conducted through the reshape2 package to uncover patterns of association among protein expressions [47]. The DESeq2 package enabled differential protein analysis with criteria established at log2FoldChange > 2 and *p*-value < 0.05; these results were subsequently visualized using ggplot2 [48,49]. To further investigate overlaps among differentially expressed proteins, a Venn diagram was created via the VennDiagram package, Version:1.7.3 [50]. Subsequently, clustering and visualization of differential proteins were carried out using the pheatmap package [51]. For a deeper understanding of the biological functions associated with these differentially expressed proteins, GO annotation along with visual assessment was performed employing R’s topGO package, version 2.56.0 [52], while KEGG annotation analyses took place on the online platform eggnog-mapper (http://eggnog-mapper.embl.de/ (accessed on 22 July 2024)). The outcomes were also illustrated using ggplot2 [48]. Finally, to further analyze the regulatory relationships among differentially expressed proteins, we extracted connections from distinct protein groups and integrated them into existing protein regulatory networks. The log2FoldChange values were derived from the differential analysis of protein sequences between alkaline and freshwater populations subjected to 7-day high alkalinity stress. These networks were constructed and subsequently visualized using Cytoscape software, version 3.10.2 [53].

## 5. Conclusions

The results indicated that a greater number of proteins in the alkaline population responded to high alkali stress, forming a complex regulatory network. Comparative analysis revealed that some of these regulatory interactions were also present in the freshwater population, including several key node genes. Notably, the alpha-globin fragment was up-regulated by hundreds or even thousands of folds in both populations in response to high alkali stress. This fragment could serve as a potential biomarker for alkali tolerance and warrants further investigation.

## Figures and Tables

**Table 1:** A comprehensive list of differentially expressed proteins at the nodes of regulatory networks shared between freshwater and alkaline populations.

Protein	Description	Alkaline Population		Freshwater Population	
		log2FoldChange	Group	log2FoldChange	Group
A0A0U2JFV2	Alpha-globin fragment	4.42	Up	9.00	Up
A0A6P6KV57	Protein phosphatase 1 regulatory subunit 3C-B-like	3.69	Up	2.13	Up
A0A6P6RIQ7	Keratin, type I cytoskeletal 13-like	2.92	Up	5.43	Up
A0A6P6K1V4	Cytidine monophosphate-N-acetylneuraminic acid hydroxylase	2.76	Up	2.41	Up
A0A6P6JC79	Apolipoprotein D	2.75	Up	2.33	Up
A0A6P6QBH9	Keratin, type I cytoskeletal 19-like	2.28	Up	3.24	Up
A0A6P6IX73	Retinol dehydrogenase 12-like	2.22	Up	2.34	Up
A0A6P6KVG5	Myeloperoxidase-like	−2.26	Down	−2.68	Down
A0A6P6RNH7	Forkhead box C1-B	−3.33	Down	−2.86	Down
A0A6P6Q9D4	Glutathione hydrolase 5 proenzyme-like	−3.83	Down	−3.30	Down
A0A6P6PY10	Sortilin-like	−5.06	Down	−2.72	Down
A0A6P6JUS2	Heme oxygenase	−5.80	Down	−5.18	Down
A0A6P6QTE8	Threonylcarbamoyl-AMP synthase	−6.48	Down	−2.77	Down
A0A6P6L830	sphingolipid 4-desaturase	−6.76	Down	−6.80	Down
A0A6P6KDG2	Xin actin-binding repeat-containing protein 2-like isoform X2	−6.84	Down	−5.70	Down
A0A6P6RC98	Leucine-rich repeat and calponin homology domain-containing protein 3-like isoform X6	−6.88	Down	−3.02	Down
A0A6P6J8K9	Brefeldin A-inhibited guanine nucleotide-exchange protein 1-like isoform X3	−7.62	Down	−2.03	Down
A0A6P6JCW0	ETS homologous factor-like	−7.86	Down	−2.55	Down
A0A6P6Q4W3	glutaminase	−3.56	Down	2.57	Up
A0A6P6PVB7	Centromere protein H-like	4.43	Up	−4.55	Down

## Data Availability

The expression levels of all proteins for all individuals have been detailed and shown in the Supplementary Materials.

## Supplementary Materials

The following supporting information can be downloaded at https://www.mdpi.com/article/10.3390/ijms252111618/s1.
